# Prevalence and Predictors of Long COVID in Patients Accessing a National Digital Mental Health Service

**DOI:** 10.3390/ijerph20186756

**Published:** 2023-09-13

**Authors:** Lauren G. Staples, Olav Nielssen, Blake F. Dear, Madelyne A. Bisby, Alana Fisher, Rony Kayrouz, Nickolai Titov

**Affiliations:** MindSpot Clinic, MQ Health, Faculty of Medicine, Health and Human Sciences, Macquarie University, Sydney, NSW 2109, Australia; olav.nielssen@mq.edu.au (O.N.); blake.dear@mq.edu.au (B.F.D.); madelyne.bisby@mq.edu.au (M.A.B.); alana.fisher@mq.edu.au (A.F.); rony.kayrouz@mq.edu.au (R.K.); nick.titov@mq.edu.au (N.T.)

**Keywords:** telehealth, service implementation, post-acute sequelae of SARS-CoV-2, coronavirus, depression, anxiety

## Abstract

MindSpot is a national mental health service that provides assessments and treatment to Australian adults online or via telephone. Since the start of 2020, questions related to the mental health impacts of COVID-19 have been routinely administered. The objective of the current study is to report the prevalence and predictors of self-reported “long COVID” in patients completing an assessment at the MindSpot Clinic between 5 September 2022 and 7 May 2023 (*n* = 17,909). Consistent with the World Health Organization definition, we defined long COVID as the occurrence of ongoing physical or mental health symptoms three months after a COVID-19 infection. We conducted a descriptive univariate analysis of patients who reported: no COVID-19 diagnosis (*n* = 6151); a current or recent (within 3 months) COVID-19 infection (*n* = 2417); no symptoms three months post-COVID-19 infection (*n* = 7468); or COVID-related symptoms at least three months post-infection (n = 1873). Multivariate logistic regression was then used to compare patients with and without symptoms three months post-COVID to identify potential predictors for long COVID. The prevalence of long COVID was 10% of the total sample (1873/17909). Patients reporting symptoms associated with long COVID were older, more likely to be female, and more likely to be depressed and report a reduced ability to perform their usual tasks. Sociodemographic factors, including cultural background, education, and employment, were examined. These results provide evidence of the significant prevalence of symptoms of long COVID in people using a national digital mental health service. Reporting outcomes in an Australian context and in specific sub-populations is important for public health planning and for supporting patients.

## 1. Introduction

The post-acute sequelae of SARS-CoV-2 or “long COVID” refers to the presence of ongoing physical or mental health symptoms for more than three months after SARS-CoV-2 (COVID-19) infection [1,2]. International estimates suggest that 10–30% of people infected with COVID-19 go on to develop some form of long COVID [3,4], while in Australia, prevalence estimates range from 5% to 10% of those infected [5]. A range of physical, cognitive, and psychological symptoms have been reported by those thought to have long COVID, including fatigue, respiratory and olfactory changes, problems with memory and concentration, anxiety, and depression [4,6]. These symptoms are often debilitating and represent a significant public health burden.

Long COVID has been associated with a range of sociodemographic, physiological, and psychological risk factors. For example, it appears to be more common in females [3,5] and may be associated with increasing age [5,7]. Higher rates have been reported for people facing socioeconomic disadvantages [3,8,9], and among people with pre-existing physical and mental health conditions [3,9,10]. However, research into the risk factors associated with long COVID is understandably limited, particularly in Australia, which had relatively low rates of COVID-19 infections until a wave of the Omicron BA.4/5 emerged during July and August 2022 [5,11].

The current study explored the prevalence and predictors of long COVID in Australian adults completing an assessment with a national government-funded digital mental health service, the MindSpot Clinic, between September 2022 and May 2023. Digital mental health services play an important public health role in delivering information and treatments online and via telephone, particularly for people who may experience difficulties accessing face-to-face care [12]. This became increasingly relevant during the COVID-19 pandemic, when many healthcare services rapidly implemented or increased the number of digital or telehealth options [13]. 

The MindSpot Clinic routinely collects sociodemographic and clinical data to ensure that services are effective, acceptable, and relevant to patients [14,15]. This means that MindSpot is in the unique position of having access to large volumes of quantitative data from patients located in all the states and territories of Australia. Since the start of the pandemic in Australia, MindSpot has collected information from patients related to their experience of and concerns about COVID-19. During the early pandemic period, we reported an increased demand for services and fluctuating levels of concern about COVID-19 [16,17,18]. We did not observe sustained increases in clinical levels of anxiety or depression in our samples, but acknowledged that any chronic consequences of COVID-19 would take time to emerge and would require ongoing monitoring and reporting. Therefore, in the current study, we aimed to examine the prevalence and predictors of self-reported chronic symptoms associated with long COVID in patients who completed an assessment with the MindSpot Clinic between September 2022 and May 2023. 

## 2. Materials and Methods

### 2.1. Participants

This uncontrolled observational cohort study included all patients who completed an assessment and answered questions related to symptoms of long COVID from 5 September 2022 to 8 May 2023. A total of 17,909 patients were included in the analysis. The patients gave consent for their de-identified data to be analyzed, and ethical approval was obtained from Macquarie University Human Research Ethics Committee (52022110184006). All patients were aged 18 years or older and eligible for Medicare-funded services.

### 2.2. Clinical Model and Study Design

The MindSpot Clinic is national digital mental health service that is funded by the Australian Department of Health and Aged Care to provide assessments of and treatment services to Australian adults with symptoms of anxiety and depression. Services are provided at no cost to the patients and are accessed via the website (www.mindspot.org.au, accessed on 23 August 2023). 

For the current study, patients provided demographic data and were asked whether they had been diagnosed with COVID-19, and if so, how long ago. Patients reporting a COVID-19 infection over three months ago were asked whether they had any continuing symptoms that they associated with a previous COVID infection, and whether the symptoms were mainly physical, cognitive, psychological, or a combination. The patients also answered the following standardized screeners to assess symptoms of depression, anxiety, and general distress using the Patient Health Questionnaire (PHQ-9), the Generalized Anxiety Disorder 7-item Scale (GAD-7), and the Kessler 10-item Scale (K10), respectively.

The PHQ-9 consists of nine items, with scores ranging from 0 to 27. Higher scores indicate higher levels of symptoms, and scores above 9 indicate a likely diagnosis of Major Depressive Disorder [19]. The GAD-7 consists of 7 items, with scores ranging from 0 to 21. The GAD-7 is sensitive to generalized anxiety disorder, panic disorder, and social anxiety, and scores over 7 indicate the likely presence of an anxiety disorder [20]. The K-10 consists of 10 items, with scores ranging from 10 to 50, and score over 20 are suggest levels of general distress that are consistent with the presence of anxiety and depressive disorders [21].

To assess the functional impact of symptoms, patients were also asked the number of whole days out of role in the previous four weeks, i.e., the number of whole days that they were totally unable to work, study, or manage their day-to-day activities because of feelings of non-specific psychological distress [22].

### 2.3. Statistical Analyses

Initial univariate analyses were conducted to compare the following groups: patients without a COVID-19 diagnosis (no COVID; *n* = 6317); patients with a recent (<3 months) or current COVID-19 infection (acute COVID; *n* = 2483); patients with no symptoms three months after a COVID-19 infection (recovered; *n* = 7662); and patients with symptoms at least three months post-COVID (long COVID; *n* = 1962). Chi-square statistics were used for categorical variables, and ANOVA with Tukey’s post hoc analyses were used for continuous variables. Bonferroni adjustment was performed for multiple comparisons. Significant variables from the univariate analyses were used in a multivariate binomial logistic regression to compare patients with and without symptoms more than three months post-COVID. An adjusted odds ratio with a 95% confidence interval was used. All analyses were conducted using SPSS version 28, and a significance level of 0.05 was used for all tests. 

## 3. Results

The univariate analyses of group differences are shown in Table 1. Univariate analyses revealed significant differences between the groups across a range of demographic characteristics, including age, gender, and marital status, and there were significant differences between groups in a range of socioeconomic factors, such as residential location, education, and income. There were also significant differences in the symptoms and health services used. The mean scores on the PHQ-9, GAD-7, K-10, and days out of role were higher for the long COVID group compared to those of all the other groups. The patients in the long COVID group were more likely to identify as female or other and were more likely to have been born outside of Australia or identify as Aboriginal or a Torres Strait Islander. 

Ten percent of the total sample (1873/17909) reported continuing symptoms that they attributed to a previous COVID-19 infection. Almost half of these patients (42.0%; 789/1873) reported a combination of physical, cognitive, and psychological symptoms. About a fifth (22.8%; 427/1873) reported mainly physical symptoms, and the remainder (35.1%; 657/1873) reported mainly psychological and cognitive impacts, most commonly, increased feelings of anxiety or depression (Table 2). 

Binomial logistic regression was performed to identify several risk factors associated with long COVID (Table 3). Age, PHQ-9 score, and the number of days out of role were significantly higher for the long COVID group compared to those of the recovered group (ORs 1.02–1.03). The other predictors were being female (OR 1.17) or another gender (OR 1.63), being born outside of Australia (OR 1.26), having a trade certificate or diploma (OR 1.28), or receiving a pension or other benefit (OR 1.29). Having a source of income that was not due to employment, pension, or a social security benefit (e.g., superannuation, investments, or trusts) was a significant protective factor (OR 0.66). 

## 4. Discussion

The symptoms associated with long COVID were reported by 10% of the total sample (1873/17909) and reported by 20% of those who were at least 3 months post-COVID infection (1873/9341). This number is higher than other Australian prevalence estimates of 5% to 10% people who previously had a COVID infection [5], but consistent with international estimates of 10–30% of people who previously had COVID infections [3,4]. Australia had relatively low levels of infection prior to late 2022, when a wave of the Omicron BA.4/5 emerged during July and August [11], and it is likely that this prevalence will increase. Given the high prevalence in the current sample of patients accessing a mental health service, the results also suggest the potentially disproportionate effect of COVID-19 on people with comorbid disorders and highlight the significant mental health challenges presented by long COVID [10,23,24].

This study found that, compared to those who had no reported history of COVID-19 infection, or had recovered, those with long COVID had more severe symptoms of depression, anxiety, and psychological distress and reported more days out of role, indicating the functional impact of the symptoms. This is unsurprising given that a range of chronic conditions are known to be associated with poorer mental health outcomes, including those that also have the potential to increase people’s susceptibility to a severe COVID-19 infection, such as cardiovascular disease [25,26,27]. Also consistent with previous research, the patients reported cognitive difficulties, such as memory loss, confusion, and difficulty concentrating [28], and future research is planned to look at how digital mental health services can address the cognitive and mental health impacts of COVID-19 in both the short term and long term. 

The factors associated with long COVID in our sample were being older, identifying as female or other, and being born outside of Australia. Other studies have shown similar factors. For example, a UK-based study of 486,149 people identified being female and part of an ethnic minority (but not increasing age) as risk factors for long COVID [9]. A recent study from The Netherlands also demonstrated a higher risk of long COVID in ethnic minority groups, highlighting the need for additional research to determine the reasons for these disparities [8]. In our study, patients with a trade certificate or diploma were also at increased risk, as were patients receiving a pension or other benefit. Conversely, having an income from superannuation, investments, trust funds, or similar, was a protective factor. Although speculative, this combination of income- and employment-related factors is likely to represent an underlying association between people’s occupational status and susceptibility to long COVID. For example, people working in front-line or essential jobs are more likely to be exposed to COVID-19, and therefore, be at greater risk of developing long COVID [5], and some research has shown that an increase in income level is significantly associated with the decreased reporting of COVID-19 symptoms [29]. Taken together, and consistent with previous research, these factors point to the complex relationship between demographic and socioeconomic circumstances and long COVID and suggest the presence of psychological and psychosocial vulnerabilities affecting recovery [8,30]. Given the increasing evidence from international research demonstrating inequalities in the social and economic burdens caused by COVID-19 [9,29,31,32], comprehensive analyses of the effects of long COVID on ethnic and other minority groups worldwide are necessary to ensure that these groups receive accessible and appropriate access to care. 

The main strength of this study is the use of a large national sample of Australian adults seeking assessments and treatment for anxiety and mood disorders. MindSpot is a high-volume national digital mental health service that provides services to Australian adults across Australia at no cost to the consumers. The proportions of many key demographic groups, including Indigenous and migrant groups, are generally representative, matching the expected proportions from national statistics [14]. As a digital service, the ability to collect large volumes of data via the online administration of standardized measurement scales also provides the advantage of being able to observe trends and benchmarks in real-world data and to use this information to help guide policy and funding decisions. However, this study does have limitations. The interactions between pre-existing symptoms of anxiety and depression and long COVID are likely to be bidirectional, and our cross-sectional, treatment-seeking sample does not necessarily provide generalizable estimates of the prevalence or risk for individuals who are not seeking mental health services. We also relied on patient-reported data for previous COVID-19 infection status and symptom information. However, there is evidence that patient-reported outcomes play an important role in identifying and managing patients with long COVID, particularly for those struggling to physically or mentally cope with their condition [6]. 

## 5. Conclusions

Despite the limitations, we observed prevalence data and risk factors that were consistent with previous research, and we considered the impact of long COVID in an Australian context. Importantly, in our study, we identified specific sub-populations of Australians seeking mental health services who may be experiencing a disproportionate burden. This information has practical application for supporting patients at an individual level and for public health planning more broadly. Long COVID is a relatively new condition in Australia, in part, because of the late spread of the virus, and the reporting of its prevalence and impact by a range of health services will help ensure informed decisions can be made by policymakers, researchers, and funders. 

## Figures and Tables

**Table 1:** Comparison of patient characteristics.

	No COVID	Acute COVID	Recovered	Long COVID	Significance
	*n* = 6151	*n* = 2417	*n* = 7468	*n* = 1873	
Age	38.0 (15.5) ^a^	35.1 (13.4) ^b^	33.2 (12.1) ^c^	35.8 (13.0) ^b^	F = 138.9; *p* < 0.001
PHQ-9	14.6 (6.5) ^a^	14.1 (6.3) ^b^	13.8 (6.3) ^b^	16.0 (6.2) ^c^	F = 65.7; *p* < 0.001
GAD-7	12.2 (5.5) ^a^	12.1 (5.3) ^a^	11.8 (5.4) ^a^	13.3 (5.2) ^b^	F = 39.3; *p* < 0.001
K-10	31.5 (8.1) ^a^	30.9 (7.9) ^b^	30.5 (7.8) ^b^	33.1 (7.7) ^c^	F = 58.1; *p* < 0.001
Days out of role	7.5 (8.8) ^a^	6.0 (7.8) ^b^	5.3 (7.2) ^c^	8.5 (8.9) ^d^	F = 122.7; *p* < 0.001
Gender					
Male	27.1% (1680) ^a^	21.2% (514) ^b,c^	22.7% (1694) ^c^	20.8% (384) ^b^	ꭙ^2^ = 72.9; *p* < 0.001
Female	71.2% (4380) ^a^	77.2% (1866) ^b^	76.2% (5689) ^b^	77.8% (1457) ^b^	
Other	1.5% (91) ^a, b^	1.5% (37) ^a,b^	1.1% (85) ^a^	1.7% (320) ^b^	
Cultural Background					
Born Aust, non-Indigenous	69.2% (4259) ^a^	72.5% (1753) ^b^	72.0% (5376) ^b^	66.6% (1248) ^c^	ꭙ^2^ = 32.6; *p* < 0.001
Indigenous Australian	4.7% (292) ^a,b^	4.4% (107) ^b^	4.3% (321) ^b^	5.8% (108) ^a^	
Born outside of Australia	26.0% (1600) ^a^	23.0% (557) ^b^	23.7% (1771) ^b^	27.6% (517) ^a^	
Residential location					
Capital city	61.0% (3750) ^a^	64.4% (1556) ^b^	64.8% (4838) ^b^	66.5% (1245) ^b^	ꭙ^2^ = 39.2; *p* < 0.001
Other urban region	20.2% (1243) ^a^	19.5% (472) ^b^	19.6% (1461) ^b^	17.2% (323) ^c^	
Rural or remote region	18.8% (1158) ^a^	16.1% (389) ^b^	15.7% (1169) ^b^	16.3% (305) ^b^	
Education					
Secondary school or below	35.9% (2207) ^a^	30.2% (731) ^b^	32.6% (2437) ^c^	31.9% (598) ^b,c^	ꭙ^2^ = 67.0 *p* < 0.001 ***
Trade certificate or diploma	27.2% (1674) ^a^	26.2% (634) ^a,b^	25.7% (1921) ^b^	30.8% (577) ^c^	
University degree	36.9% (2270) ^a^	43.5% (1052) ^b^	41.6% (3110) ^b^	37.3% (698) ^a^	
Income					
Employed full or part time	57.0% (3504) ^a^	71.5% (1728) ^b^	73.3% (5475) ^b^	63.4% (1188) ^c^	ꭙ^2^ = 522.4 *p* < 0.001 ***
Unemployed, no income	14.7% (906) ^a^	11.5% (277) ^b^	11.9% (887) ^b^	14.0% (262) ^a^	
Pension or benefit	21.8% (1342) ^a^	12.6% (305) ^b^	10.9% (811) ^c^	19.4% (364) ^d^	
Other source of income	6.5% (399) ^a^	4.4% (107) ^b^	4.0% (295) ^b, c^	3.2% (59) ^c^	
Marital status					
Never married	51.3% (3153) ^a,b^	50.5% (1221) ^b^	52.8% (3944) ^a^	46.6% (872) ^c^	ꭙ^2^ = 136.8 *p* < 0.001 ***
Married	31.3% (1924) ^a^	35.7% (863) ^b^	35.8% (2675) ^b^	35.9% (673) ^b^	
Divorced, separated, or widowed	17.5% (1074) ^a^	13.8% (333) ^b^	11.4% (849) ^c^	17.5% (328) ^a^	
Mental Health Professional use					
None	32.8% (2018) ^a^	34.2% (826) ^a^	37.3% (2789) ^b^	29.3% (548) ^c^	ꭙ^2^ = 63.1 *p* < 0.001 ***
Previous	45.8% (2815) ^a^	45.3% (1095) ^a,b^	43.7% (3260) ^b^	47.1% (882) ^a^	
Current	21.4% (1318) ^a^	20.5% (496) ^a,b^	19.0% (1419) ^b^	23.7% (443) ^c^	

Continuous variables are shown as mean (SD). Categorical variables are shown as % (*n*). Each superscript letter in each row denotes a subset whose column proportions do not differ significantly from each other using chi-square univariate analysis. *** *p* is significant at < 0.001.

**Table 2:** Main cognitive or psychological impact of long COVID.

	Mainly Cognitive or Psychological Impact
	*n* = 657
Increased anxiety or worry	35.3% (232)
Increased depression or low mood	29.4% (193)
Memory loss, confusion, or difficulties concentrating	14.0% (92)
Increased loneliness or isolation	12.0% (81)
Increased use of alcohol or other substances	4.3% (28)
Other	4.7% (31)

**Table 3:** Predictors of long COVID.

	*p*	Adjusted Odds Ratio	95% CI for Exp (B)	
		Exp (B)	Lower	Upper
Age	<0.001	1.021	1.017	1.026
Depression (PHQ-9)	0.003	1.030	1.014	1.046
Days out of role	<0.001	1.028	1.020	1.036
Anxiety (GAD-7)	0.272			
General distress (K-10)	0.100			
Gender	0.023			
Male	Reference			
Female	0.024	1.166	1.020	1.333
Other	0.039	1.628	1.024	2.590
Cultural Background	0.001			
Born Aust, non-Indigenous	Reference			
Indigenous Australian	0.311	1.135	0.896	1.449
Born outside of Australia	<0.001	1.264	1.113	1.435
Education	0.002			
Secondary school or below	Reference			
Trade certificate or diploma	<.001	1.286	1.116	1.481
University degree	0.157	1.108	0.962	1.276
Income	<0.001			
Employed full or part-time	Reference			
Unemployed, no income	0.119	1.145	0.966	1.359
Pension or benefit	0.004	1.281	1.084	1.514
Other source of income	0.007	0.659	0.486	0.894
Residential location	0.116			
Marital status	0.420			
Mental health professional use	0.201			

## Data Availability

Access to de-identified data may be provided to external researchers upon reasonable request. Requests must be made in writing and are subject to the establishment of appropriate data governance and the approval of an independent and recognized Human Research Ethics Committee.
